# A Novel Glutamyl (Aspartyl)-Specific Aminopeptidase A from *Lactobacillus delbrueckii* with Promising Properties for Application

**DOI:** 10.1371/journal.pone.0152139

**Published:** 2016-03-22

**Authors:** Timo Stressler, Jacob Ewert, Michael Merz, Joshua Funk, Wolfgang Claaßen, Sabine Lutz-Wahl, Herbert Schmidt, Andreas Kuhn, Lutz Fischer

**Affiliations:** 1 Department of Biotechnology and Enzyme Science, Institute of Food Science and Biotechnology, University of Hohenheim, Stuttgart, Germany; 2 Department of Food Microbiology and Hygiene, Institute of Food Science and Biotechnology, University of Hohenheim, Stuttgart, Germany; 3 Institute of Microbiology, University of Hohenheim, Stuttgart, Germany; National University of Ireland—Galway, IRELAND

## Abstract

Lactic acid bacteria (LAB) are auxotrophic for a number of amino acids. Thus, LAB have one of the strongest proteolytic systems to acquit their amino acid requirements. One of the intracellular exopeptidases present in LAB is the glutamyl (aspartyl) specific aminopeptidase (PepA; EC 3.4.11.7). Most of the PepA enzymes characterized yet, belonged to *Lactococcus lactis* sp., but no PepA from a *Lactobacillus* sp. has been characterized so far. In this study, we cloned a putative *pepA* gene from *Lb*. *delbrueckii* ssp. *lactis* DSM 20072 and characterized it after purification. For comparison, we also cloned, purified and characterized PepA from *Lc*. *lactis* ssp. *lactis* DSM 20481. Due to the low homology between both enzymes (30%), differences between the biochemical characteristics were very likely. This was confirmed, for example, by the more acidic optimum pH value of 6.0 for *Lb*-PepA compared to pH 8.0 for *Lc*-PepA. In addition, although the optimum temperature is quite similar for both enzymes (*Lb*-PepA: 60°C; *Lc*-PepA: 65°C), the temperature stability after three days, 20°C below the optimum temperature, was higher for *Lb*-PepA (60% residual activity) than for *Lc*-PepA (2% residual activity). EDTA inhibited both enzymes and the strongest activation was found for CoCl_2_, indicating that both enzymes are metallopeptidases. In contrast to *Lc*-PepA, disulfide bond-reducing agents such as dithiothreitol did not inhibit *Lb*-PepA. Finally, *Lb*-PepA was not product-inhibited by L-Glu, whereas *Lc*-PepA showed an inhibition.

## Introduction

Lactic acid bacteria (LAB) are a heterogeneous group of microorganisms which have a common metabolic property: the production of lactic acid as the majority end-product from the fermentation of carbohydrates [1,2]. The LAB are commonly Gram-positive, aerobic to facultative anaerobic, asporogenous roods and cocci, which are oxidase, catalase and benzidine negative [1]. They are usually mesophilic, but can grow at temperatures as low as 5°C or as high as 45°C [3]. The majority of strains grow at pH 4.0–4.5, however, some are active at pH 9.6 and others at pH 3.2 [3]. In general, LAB members are nonpathogenic organisms with the reputable Generally Recognized as Safe (GRAS) status [2]. Typical LAB species belong to the genera *Lactobacillus*, *Lactococcus*, *Streptococcus*, *Pediococcus*, *Oenococcus*, *Enterococcus*, and *Leuconostoc* [1]. Depending on the species and on the strain, LAB are auxotrophic for a variable number of amino acids. The genome of *Lb*. *plantarum* WCFS1, for instance, encodes enzymes for the biosynthesis of most amino acids, except valine, leucine and isoleucine [4]. By comparison, based on the *in silico* analyses, *Lb*. *acidophilus* NCFM is likely to be auxotrophic for 14 amino acids [5]. Therefore, LAB require a fully active proteolytic system to acquit their amino acid requirements [2] and, thus, LAB are a rich source for proteolytic enzymes. The proteolytic system of LAB can be divided into three groups on the basis of their function [2,6]: (i) extracellular peptidases, which break proteins into peptides; (ii) transport systems, which translocate the resulting products across the cytoplasm membrane and, (iii) intracellular peptidases, which degrade peptides into smaller peptides and amino acids. The intracellular peptidases have distinct, but overlapping activities [6], and they can be divided into endopeptidases and exopeptidases. The endopeptidase PepO, for example, was the first endopeptidase characterized from a LAB species and is capable of hydrolyzing oligopeptides, but unable to hydrolyze casein itself [7]. The exopeptidases are classified by their specificity: for instance, the general aminopeptidase PepN (EC 3.4.11.2) [8], the proline specific peptidases, such as PepX (EC 3.4.14.11) [8,9] and PepP (EC 3.4.11.9) [10], or the glutamyl (aspartyl) specific aminopeptidase PepA (EC 3.4.11.7) [11]. The PepA is a metal-dependent enzyme and specific for peptides with the N-terminal amino acids glutamic acid, aspartic acid and serine [11]. A possible application of PepA is the hydrolysis of glutamyl/aspartyl-rich food proteins. Examples of such proteins are wheat gluten [12] and casein [13]. However, due to the fact that PepA is an exopeptidase, a prehydrolysis of the particular protein source with an endopeptidase preparation such as Alcalase^®^ (Novozymes) will be necessary. A combination of an endopeptidase with PepA and other exopeptidases will probably result in a higher degree of hydrolysis, because the synergy of endopeptidases and exopeptidases is the key for an efficient hydrolysis of proteins [14]. The synergy between two exopeptidases was shown for a casein hydrolysis from Stressler et al. [8] for the general aminopeptidase N (PepN) and the proline-specific X-prolyl-dipeptidyl aminopeptidase (PepX). The additional usage of a protein glutaminase (EC 3.5.1.44) will probably result in an increase of PepA substrates due to the biotransformation of Gln to Glu. A further advantage of this biotransformation will be an increased solubility of the protein (e.g. wheat gluten) [15], which increases the productivity of the process. A high degree of hydrolysis is preferable for the production of flavoring hydrolysates. An increased amount of Glu/Asp in food protein hydrolysates is especially desirable, due to its umami taste profile [16]. Hydrolysates of wheat gluten, for example, are used for delivering savory taste (umami) in a wide range of culinary products [17,18]. Concerning PepA from LAB, only a few articles exist, mainly on PepA from *Lactococcus lactis* sp., and were published between 1987 and 1995 [11,19–21]. In 2010, an article was published concerning the substrate specificity and the metal-binding site of PepA from *Streptococcus pneumonia* R6 [22]. To the best of our knowledge, no article is available which deals with the PepA from a *Lactobacillus* sp. However, there is a gene present in the genome of *Lb*. *delbrueckii* sp. *lactis* DSM 20072 which could encode for PepA (UniProt ID: F0HXE4), but no gene/function relationship has yet been attributed. The gene homology, in comparison to the *pepA* gene from *Lc*. *lactis* ssp. *cremoris* MG1316 (UniProtID: Q48677; reviewed), is 26%. The homology is 30% based on the translated amino acid sequence. These homologies are quite low in comparison to the gene homology of 85% and the protein homology of 94% between *Lc*. *lactis* ssp. *cremoris* MG1316 (UniProt ID: Q48677; reviewed) and *Lc*. *lactis* ssp. *lactis* IL1403 (UniProt ID: Q9CIH3; unreviewed), respectively. Due to the low homology between the known PepA from *Lc*. *lactis* sp. and the heretofore unknown PepA from *Lb*. *delbrueckii*, it is very likely that the biochemical characteristics are quite different. Thus, the aim of the current study was the cloning and heterologous recombinant production of the PepA from *Lb*. *delbrueckii* ssp. *lactis* DSM 20072 and its biochemical characterization. Furthermore, the characteristics were compared directly with the characteristics of PepA from *Lc*. *lactis* ssp. *lactis* DSM 20481, which was also heterologously recombinantly produced in *Escherichia coli* BL21(DE3).

## Materials and Methods

### Chemicals, enzymes, kits, materials and devices

All chemicals were of analytical grade and purchased from Sigma Aldrich (Taufkirchen, Germany), Carl Roth GmbH (Karlsruhe, Germany) or Applichem (Darmstadt, Germany). Hexokinase/glucose-6-phosphate dehydrogenase was purchased from Megazyme International Ireland (Wicklow, Irland) and was used for the glucose concentration determination assay based on the commercial d-glucose/d-fructose test kit from R-Biopharm AG (Darmstadt, Germany; product code 10 139 106 035). Chromogenic peptides were obtained from Bachem AG (Bubendorf, Switzerland). Molecular weight markers were bought from New England Biolabs (NEB; Frankfurt, Germany) and GE Healthcare (München, Germany). The enzymes required for molecular biological work were purchased from NEB (Frankfurt, Germany), Qiagen (Hilden, Germany), Thermo Scientific (Schwerte, Germany) or Roche Applied Science (Penzberg, Germany). Kits for molecular biological work were obtained from Thermo Scientific (Schwerte, Germany) or Qiagen (Hilden, Germany). Agarose was bought from SERVA Electrophoresis GmbH (Heidelberg, Germany). PD-10 columns were obtained from GE Healthcare (München, Germany). The bioreactor cultivation was realized using the Multifors system (Infors AG, Bottmingen/Basel, Switzerland). The MINI-PROTEAN system (Bio-Rad Laboratories GmbH, München, Germany) was used for polyacrylamide gel electrophoresis. The ÄKTA-FPLC system (GE Healthcare, München, Germany) equipped with a Ni-NTA column (Cube Biotech GmbH, Monheim, Germany) was used for protein purification.

### Bacterial strains

*Lactobacillus delbrueckii* ssp. *lactis* DSM 20072 was cultivated in de Man, Rogosa and Sharpe (MRS) medium [23] with constant shaking at 37°C. *Lactococcus lactis* ssp. *lactis* DSM 20481 was cultivated as described previously [10]. *Escherichia coli* XL1 Blue (Merck, Darmstadt, Germany) and *E*. *coli* BL21(DE3) (Novagen, Madison, USA) were used as the hosts for the cloned polymerase chain reaction (PCR) products and T7 expression work, respectively. Standard protocols were employed for the preparation and transformation of competent *E*. *coli* cells with plasmid DNA via heat shock [24]. The *E*. *coli* cells were cultivated as described previously [8,10].

### Cloning, construction of expression vectors and sequencing

Total genomic DNA from either *Lb*. *delbrueckii* or *Lc*. *lactis* was extracted using an identical method to that described previously [10]. The PCR was performed using HotStar HiFidelity polymerase (Qiagen), according to the manufacturer’s instructions. All primers used in this study were synthesized by biomers.net GmbH (Ulm, Germany). The primers *Lb*_*pepA*_for (5´-GTGACGAACATATGGAAAAAGCCGCTGAAATTC-3´; *Nde*I restriction site is underlined) and *Lb*_*pepA*_rev (5´-CGGAGCTCGAGATTAAAGCTTTTAAAGGATTCCAGCTTTTC-3´; *Xho*I restriction site is underlined) were used for the amplification of the *pepA* gene from *Lb*. *delbrueckii* ssp. *lactis* DSM 20072 (UniProt ID: F0HXE4; EMBL: EGD26747). The primers *Lc*_*pepA*_for (5´-GCCGCCGCATATGGAACTATTCGACAAAG-3´; *Nde*I restriction site is underlined) and *Lc*_*pepA*_rev (5´-CGGAGCCGCTCGAGATAGTTTTTAATTTCAGCTAC-3´; *Xho*I restriction site is underlined) were used for the amplification of the *pepA* gene from *Lc*. *lactis* ssp. *lactis* DSM 20481. The last two primers were designed based on the nucleotide sequence of the *pepA* gene from *Lc*. *lactis* ssp. *lactis* (strain IL1403; UniProt ID: Q9CIH3; EMBL: AAK04485).

The PCR products obtained (about 1100 bp) of the *Lb-pepA* gene (1086 bp) and the *Lc-pepA* gene (1068 bp) were purified using the QIAquick PCR Purification kit (Qiagen) according to the manufacturer’s instructions. The purified PCR products and the vector pET20b(+) (Novagen) were digested using the restriction enzymes *Nde*I and *Xho*I. T4-DNA-ligase was used for ligation of the digested PCR products and vector and resulted in the plasmids pET20b(+)_*Lb-pepA* and pET20b(+)_*Lc-pepA*, respectively. Both vectors were individually transformed into competent *E*. *coli* XL1 Blue cells via heat shock and plated on LB_amp_ agar plates (tryptone: 10 g L^-1^, yeast extract: 5 g L^-1^, NaCl: 5 g L^-1^, ampicillin: 100 μg mL^-1^). After cultivation overnight at 37°C, single colonies were picked and cultivated in 5 mL LB_amp_ medium overnight at 37°C. The plasmids were isolated using the GeneJET Plasmid Miniprep kit (Fermentas), according to the manufacturer’s instructions. The plasmids obtained were used for sequencing (SRD–Scientific Research and Development GmbH; Bad Homburg, Germany). Database searches and alignments were performed online with the programs blastn and blastp provided by the BLAST server [25,26]. All parameters were set at their default values.

### Expression of recombinant PepA in *E*. *coli* BL21(DE3)

Transformed *E*. *coli* BL21(DE3) strains were grown in 2xYT medium (tryptone: 16 g L^-1^, yeast extract: 10 g L^-1^, NaCl: 5 g L^-1^) that contained glucose (10 g L^-1^) supplemented with ampicillin (100 μg mL^-1^). Pre-cultures were incubated at 37°C on a rotary shaker. The first (5 mL) and the second pre-culture (50 mL) were each cultivated for 15 h. The main cultures (600 mL) were grown in a parallel bioreactor system (Multifors) and inoculated with 10% (v/v) of the particular pre-culture. The pH value of the bioreactor cultivation was kept at pH 7.0 by using 2 M NaOH and 2 M H_3_PO_4_. The O_2_ concentration (pO_2_) dissolved in the medium was maintained above 30% saturation by regulation of the stirrer speed (500–1000 rpm). The aeration rate was 1 vvm. Samples were taken during the cultivation to analyze the optical density (OD_600nm_), the bio dry mass (BDM) and the glucose concentration, as described previously [10]. When the OD_600nm_ value reached 5, the temperature was maintained at 30°C to minimize the formation of inclusion bodies, and recombinant protein expression was induced by the addition of 0.5 mM isopropyl *β*-d-1-thiogalactopyranoside (IPTG). The cultures were harvested after 11 h of cultivation, as described previously [10] and then stored at -20°C.

Samples (10 mL) were taken at various time points during the cultivations, and the PepA activity (see below) was determined from the cell-free extract after cell disruption, centrifugation (8000 x *g*, 10 min, 4°C) and filtration (0.45 μm). Consequently, the samples were centrifuged (see above) and the cell-pellets were suspended in a 0.9% (w/v) NaCl-solution (5 mL). After centrifugation (see above), the cell-pellets were suspended in Na/K-phosphate (50 mM; pH 6.0 for *Lb*-PepA or pH 8.0 for *Lc*-PepA). Subsequently, the cell disruption was realized by sonification (UP200S ultrasonic processor, Dr. Hielscher, Berlin, Germany; 20 cycles containing 1 min disruption, 1 min break) on ice.

*E*. *coli* BL21(DE3)_pET20b(+) (reference without recombinant PepA) was cultivated under the same conditions for comparison.

### Purification of PepA

Both *Lb*-PepA and *Lc*-PepA were purified individually by Ni^2+^-NTA chromatography using an ÄKTA-FPLC system (GE Healthcare). At first, cell suspensions (15% (w/v)) in binding buffer (B1: 50 mM Tris/HCl + 500 mM NaCl + 40 mM imidazole, pH 8.0 for *Lb*-PepA; B2: 50 mM Na/K-phosphate + 500 mM NaCl + 40 mM imidazole, pH 8.0 for *Lc*-PepA) were prepared for cell disruption (see above). The supernatants (cell-free extract) after centrifugation (8000 x *g*, 20 min, 4°C) and filtration (0.45 μm) were applied to the Ni^2+^-NTA column (Cube Biotech GmbH, Monheim, Germany; 1 column volume (CV) = 5 mL). Usually, 10 mL of the particular cell-free extract was injected at a flow rate of 1 mL min^-1^ using binding buffer B1 or B2. Afterwards, the unbound protein was washed out for 10 CV. Bound protein was eluted with elution buffer (E1: 50 mM Tris/HCl + 500 mM NaCl + 500 mM imidazole, pH 8.0 for *Lb*-PepA; E2: 50 mM Na/K-phosphate + 500 mM NaCl + 500 mM imidazole, pH 8.0 for *Lc*-PepA) by a linear gradient (4 CV) to 100% elution buffer at a flow rate of 1 mL min^-1^ and detected at 280 nm. Fractions (3.5 mL) were collected and PepA-active fractions were pooled, desalted into Na/K-phosphate buffer (50 mM; pH 6.0 for *Lb*-PepA or pH 8.0 for *Lc*-PepA) using PD-10 columns (GE Healthcare) and stored at -20°C until use.

### Polyacrylamide gel electrophoresis (PAGE)

Samples were divided into soluble and insoluble fractions after cell disruption. These fractions and purified PepA (also soluble) were analyzed by sodium dodecyl sulfate (SDS) PAGE (12.5% gel) [27]. An amount of 5 μg protein [28] or 7.5 μg protein [29] was applied to gel in the case of the soluble or insoluble fractions, respectively. Bovine serum albumin was used as a standard for both protein determination methods [28,29]. A commercial molecular weight protein mixture was used as a reference for molecular weight estimation (NEB; 2–212 kDa). Gels were stained with Coomassie Brilliant Blue for protein detection.

### Size exclusion chromatography (SEC)

The native molecular mass determination of the purified enzymes by SEC was realized using an ÄKTA-FPLC system (GE Healthcare) equipped with a Superdex^TM^ 200 10/300 GL column (GE Healthcare). The injection volume was 30 μL (1 mg_Protein_ mL^-1^) and the flow rate was 0.75 mL min^-1^ using Na/K-phosphate buffer (10 mM; pH 6.0 for *Lb*-PepA or pH 8.0, for *Lc*-PepA) containing NaCl (150 mM) as the eluent. Eluted protein was detected at 280 nm and fractions (0.5 mL) were taken. Standard proteins (Gel Filtration HMW and LMW Calibration Kit, GE Healthcare) were used as references for molecular mass determination.

### Standard PepA enzyme activity assay

PepA activity in the standard assay was determined with H-Asp-*p*NA as a substrate. The standard assay was performed as follows: Initially, 25 μL enzyme solution was added to 192.5 μL Na/K-phosphate buffer (50 mM; pH 6.0 for *Lb*-PepA or pH 8.0 for *Lc*-PepA). Additionally, 10 μL of a CoCl_2_ stock solution (15 mM for *Lb*-PepA or 7.5 mM for *Lc*-PepA) was added. After incubation for 10 min at the required temperature (60°C for *Lb*-PepA or 65°C for *Lc*-PepA), 12.5 μL of the substrate solution (5 mg mL_DMSO_^-1^) was added to the reaction mixture. The reaction was terminated by adding 50 μL acetic acid (50% (v/v) to the sample. After centrifugation (8000 x *g*, 5 min, 4°C), 240 μL of the supernatant was transferred into a microtiter plate and the absorption was measured (Multiskan FC, Thermo Scientific, Braunschweig, Germany) at 405 nm. One katal (kat) of PepA activity was defined as the release of 1 mol *p*-nitroanilin per s. The specific activity of a particular sample was determined by dividing the volumetric activity by the corresponding protein content [28]. Thus, the specific activity during the bioreactor cultivation is referred to the protein content in the supernatant after cell disruption. All other specific activity values are related to the enzymes after purification.

### Characterization of *Lb*-PepA and *Lc*-PepA

The purified *Lb*-PepA and *Lc*-PepA were characterized. The standard assay with H-Asp-*p*NA as a substrate was used unless stated otherwise.

#### Influence of temperature and pH on the initial PepA activity

In contrast to the standard assay, the temperature varied between 10 and 80°C. The pH was varied in the range between pH 4.5 and 10.0 (depending on the enzyme tested) for determination of the pH-dependent effect. All buffers had a concentration of 50 mM. The following buffers were used for *Lb*-PepA: Na-acetate/acetic acid (pH 5.0–6.0), Na/K-phosphate (pH 5.5–8.0) and Bis-Tris-propane/HCl (pH 6.0–7.0). The following buffers were tested for *Lc*-PepA: Na/K-phosphate (pH 6.0–8.0), Tris/HCl (pH 7.5–8.5) and Glycine/NaOH (pH 8.5–10.0).

#### Temperature stability of PepA

The enzyme preparations were incubated at 0, 40, 50 and 60°C (*Lb*-PepA) or at 0, 45, 55 and 65°C (*Lc*-PepA) for up to three days for the temperature stability and samples were taken several times. Sodium azide (0.1% (w/v)) was added to prevent microbial growth.

#### Storage stability of PepA

The storage stability of PepA was determined for four different storage conditions: (i) aliquotes (20 μL) of the enzyme solutions were stored at -80°C; (ii) aliquotes (20 μL) were lyophilized and stored in a desiccator at 20°C; (iii) aliquotes (20 μL) were lyophilized and stored at -80°C; and (iv) aliquotes (300 μL) were stored at -80°C, but were frozen again after thawing. Several samples were taken during the storage time (two months) and the PepA activity was measured.

#### Influence of metal ions on the PepA activity

The influence of different metal ions (CoCl_2_, MnCl_2_, ZnCl_2_) on the activity of purified PepA (1.2 ± 0.3 mg_Protein_ mL^-1^) was analyzed. In addition, apo-PepA was prepared by treating purified PepA with 20 mM ethylenediaminetetraacetic acid (EDTA), followed by dialysis in Na/K-phosphate buffer (50 mM; pH 6.0 for *Lb*-PepA or pH 8.0 for *Lc*-PepA). Subsequently, the reactivation of apo-PepA was tested for CoCl_2_, MnCl_2_, ZnCl_2_, CaCl_2_ and MgCl_2_. In contrast to the standard assay, the concentration of these substances in the final assay varied between 0.04 and 5 mM.

#### Influence of organic solvents, inhibitors and other reagents

The substances tested were dissolved in H_2_O_dd_, DMSO, acetone or ethanol, depending on the substance. The assay conditions were identical to the standard assay, except that 10 μL of the test substance and 182.5 μL buffer were used, instead of 192.5 μL buffer. The concentration of the inhibitors, metal chelators, reducing agents and other substances in the final assay varied between 0.001 and 10 mM.

#### Determination of the substrate specificity of PepA

The substrate specificity of PepA was determined with different chromogenic substrates. The following *p*NA-derivates were used in a concentration of 5 mg mL_DMSO_^-1^: H-Asp-*p*NA, H-Glu-*p*NA, H-Ala-*p*NA, H-Gly-*p*NA, H-Ile-*p*NA, H-Val-*p*NA, H-Pro-*p*NA, H-Leu-*p*NA, H-His-*p*NA, H-Phe-*p*NA, H-Arg-*p*NA, and H-Lys-*p*NA.

#### Determination of product inhibition of PepA

The product inhibition of PepA was tested for the single amino acids L-Asp and L-Glu in a final concentration of 0.1, 1 and 10 mM. The assay conditions were identical to the standard assay, except that 10 μL of the particular amino acid solutions and 182.5 μL buffer were used, instead of 192.5 μL buffer.

#### Determination of apparent kinetic parameters of PepA

The apparent kinetic parameters of PepA were determined using H-Asp-*p*NA and H-Glu-*p*NA as a substrate. Standard PepA activity assay conditions were used in which the final substrate concentration ranged from 0.06–18 mM, depending on the particular enzyme and substrate. In addition, the apparent kinetic parameters of reactivated apo-*Lb*-PepA were investigated. In contrast to the standard assay, a particular metal salt stock solution was added to apo-*Lb*-PepA to gain the optimal metal concentration, as determined previously. The results were plotted according to Michaelis-Menten and the apparent kinetic parameters were calculated by nonlinear regression fitting using SigmaPlot 12.5 (Systat Software, Inc., San Jose, CA).

### Statistical analysis

Standard deviation was used for data evaluation and calculated with Excel (Microsoft, Redmond, USA). All experiments were conducted at least in duplicate, with three independent measurements. The standard deviation was always below 5%.

## Results

In this study, the heretofore unknown PepA from *Lb*. *delbrueckii* ssp. *lactis* DSM 20072 was produced recombinantly and compared to the biochemical characteristics of PepA from *Lc*. *lactis* ssp. *lactis* DSM 20481. Due to the low homology between the two enzymes (see below), it is very likely that the biochemical characteristics will be different.

### Cloning and sequencing of *Lb-pepA* and *Lc-pepA*

The *Lb*-PepA expression vector (pET20b(+)_*Lb-pepA*) and the *Lc*-PepA expression vector (pET20b(+)_*Lc-pepA*) were constructed, sequenced and used for individual expression in the *E*. *coli* BL21(DE3) host strain. Due to the cloning strategy chosen, the proteins produced contained a C-terminal His_6_-tag. The nucleotide sequence of both the *Lb-pepA* gene (source: *Lb*. *delbrueckii* ssp. *lactis* DSM 20072) and the *Lc-pepA* gene (source: *Lc*. *lactis* ssp. *lactis* DSM 20481) obtained in this study exhibited 100% identity to sequences of *pepA* genes from *Lb*. *delbrueckii* ssp. *lactis* DSM 20072 (UniProt ID: F0HXE4; EMBL: EGD26747) and *Lc*. *lactis* ssp. *lactis* CV56 (UniProt ID: F2HIS5; EMBL: ADZ63034), respectively, deposited previously. By comparison, an identity of about 30% was ascertained for the amino acid sequences of both PepA enzymes used in this study.

### Heterologous expression of *Lb*-PepA and *Lc*-PepA in *E*. *coli*

**Fig 1:**
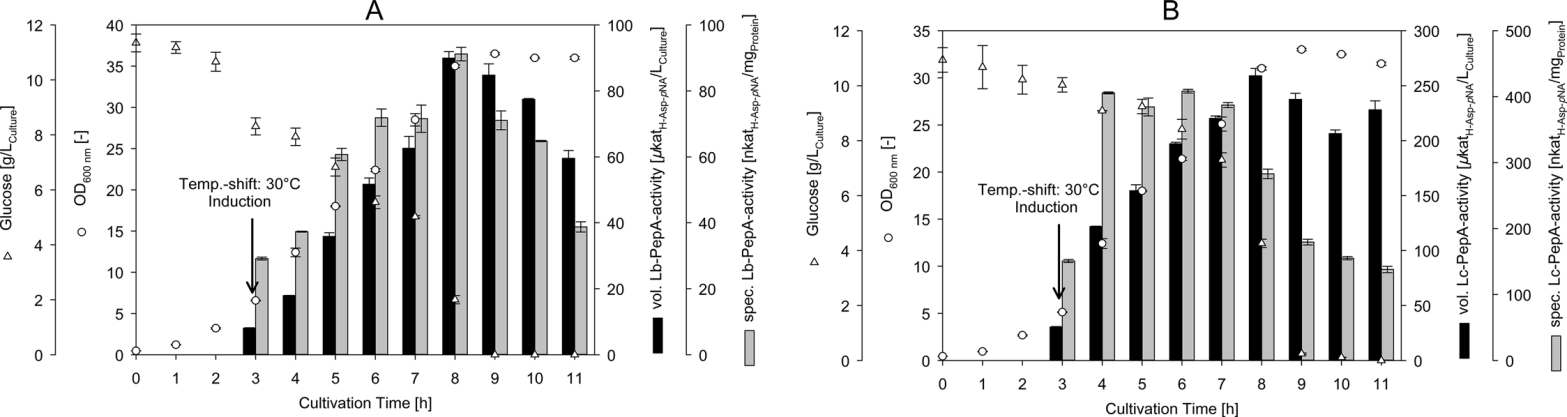
**Course of the bioreactor cultivation (working volume: 600 mL) of recombinant *E*. *coli* BL21(DE3) for the production of *Lb*-PepA (A) and *Lc*-PepA (B).** Cultivations began at 37°C and shifted to 30°C with simultaneous induction using IPTG (see arrow). The means ± standard deviation of three independent measurements are presented.

Both *Lb*-PepA and *Lc*-PepA were individually expressed in soluble form using the expression host *E*. *coli* BL21(DE3) under identical cultivation conditions (Fig 1). During the cultivation of the recombinant *E*. *coli* for *Lb*-PepA production, the glucose was completely consumed after approximately 9 h when the cells entered the stationary growth phase (Fig 1A). The OD_600 nm_ value increased up to 36 during cultivation (corresponding to a cell dry weight of 10.3 g L^-1^), and the maximum volumetric *Lb*-PepA activity was achieved after 8 h of cultivation with about 90 μkat_H-Asp-pNA_ L_Culture_^-1^ (specific *Lb*-PepA activity: 92 nkat_H-Asp-pNA_ mg_Protein_^-1^). The *Lb*-PepA activity decreased during the stationary growth phase, which was caused by degradation of the enzyme, as seen on the SDS-PAGE analysis (data not shown).

In the case of *Lc*-PepA production, a comparable maximum OD_600 nm_ value of 33 was achieved with the *E*. *coli* expression host (Fig 1B). This corresponds to a cell dry weight of 9.4 g L^-1^. Again, the glucose was consumed after approximately 9 h by entering the stationary growth phase. The maximum volumetric *Lc*-PepA activity (260 μkat_H-Asp-pNA_ L_Culture_^-1^) was determined after 8 h of cultivation and decreased during the stationary growth phase. The highest specific *Lc*-PepA activity (410 nkat_H-Asp-pNA_ mg_Protein_^-1^) was determined after 6 h of cultivation and also decreased with a longer cultivation time.

Analysis of the samples taken during the cultivation by SDS-PAGE showed that both enzymes were present in both soluble and insoluble form (inclusion bodies) in a ratio of approximately 1:1.

For comparison, *E*. *coli* BL21(DE3) transformed with the insert-free pET20b(+) vector was cultivated under identical conditions to determine the background PepA activity of the expression host. No PepA activity was measured at any time during the cultivation.

### Purification of *Lb*-PepA and *Lc*-PepA and molecular mass determination

**Fig 2:**
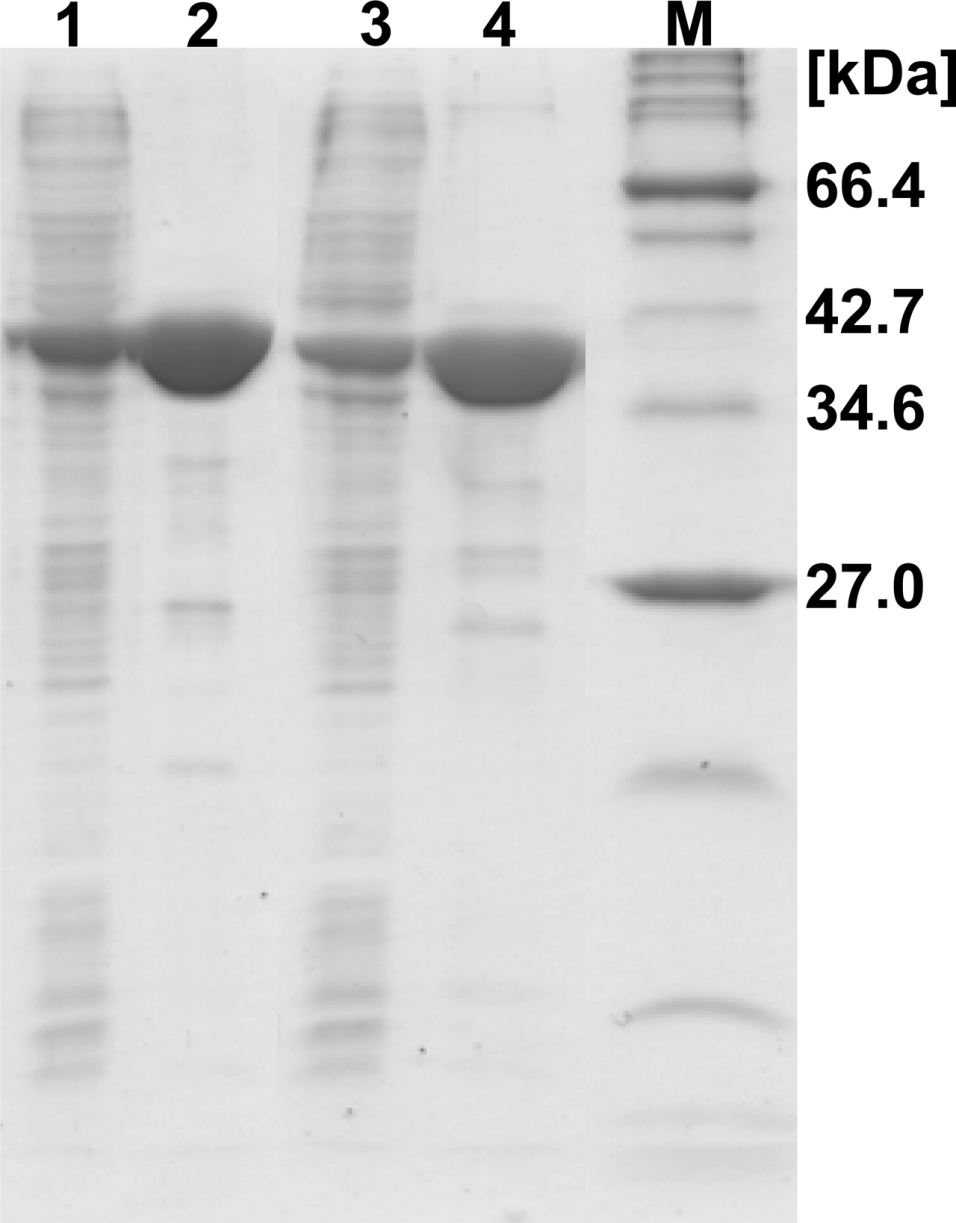
SDS-PAGE before (lane 1 and 3) and after (lane 2 and 4) Ni^2+^ affinity purification of *Lb*-PepA (lane 1 and 2) and *Lc*-PepA (lane 3 and 4). The molecular mass marker is presented in lane M.

The His_6_-tagged enzymes *Lb*-PepA and *Lc*-PepA were both individually purified using a FPLC procedure based on Ni^2+^-NTA chromatography resin. An enzymatic activity yield of approximately 30% was achieved for each enzyme, whereas a purification factor of 2.8 and 7.1 was determined for *Lb*-PepA and *Lc*-PepA, respectively. The purity and the molecular mass of the monomers were determined by SDS-PAGE (Fig 2). The molecular mass of the *Lb*-PepA monomer was determined at approximately 41 kDa (Fig 2, lane 1 and 2). This is in accordance with the theoretical molecular mass of 41.1 kDa (based on the amino acid sequence including the His_6_-tag). A molecular mass of approximately 40 kDa was determined for *Lc*-PepA (Fig 2, lane 3 and 4). The theoretical molecular mass of *Lc*-PepA (including the His_6_-tag) is 39.4 kDa. Size exclusion chromatography experiments were performed to analyze the native mass of the enzymes, and a molecular mass of 508 kDa and 470 kDa was determined for *Lb*-PepA and *Lc*-PepA, respectively. By taking the molecular mass of the monomers into account, it is suggested that both enzymes are homo dodecamers.

### Effect of temperature and pH on the initial PepA activity

**Fig 3:**
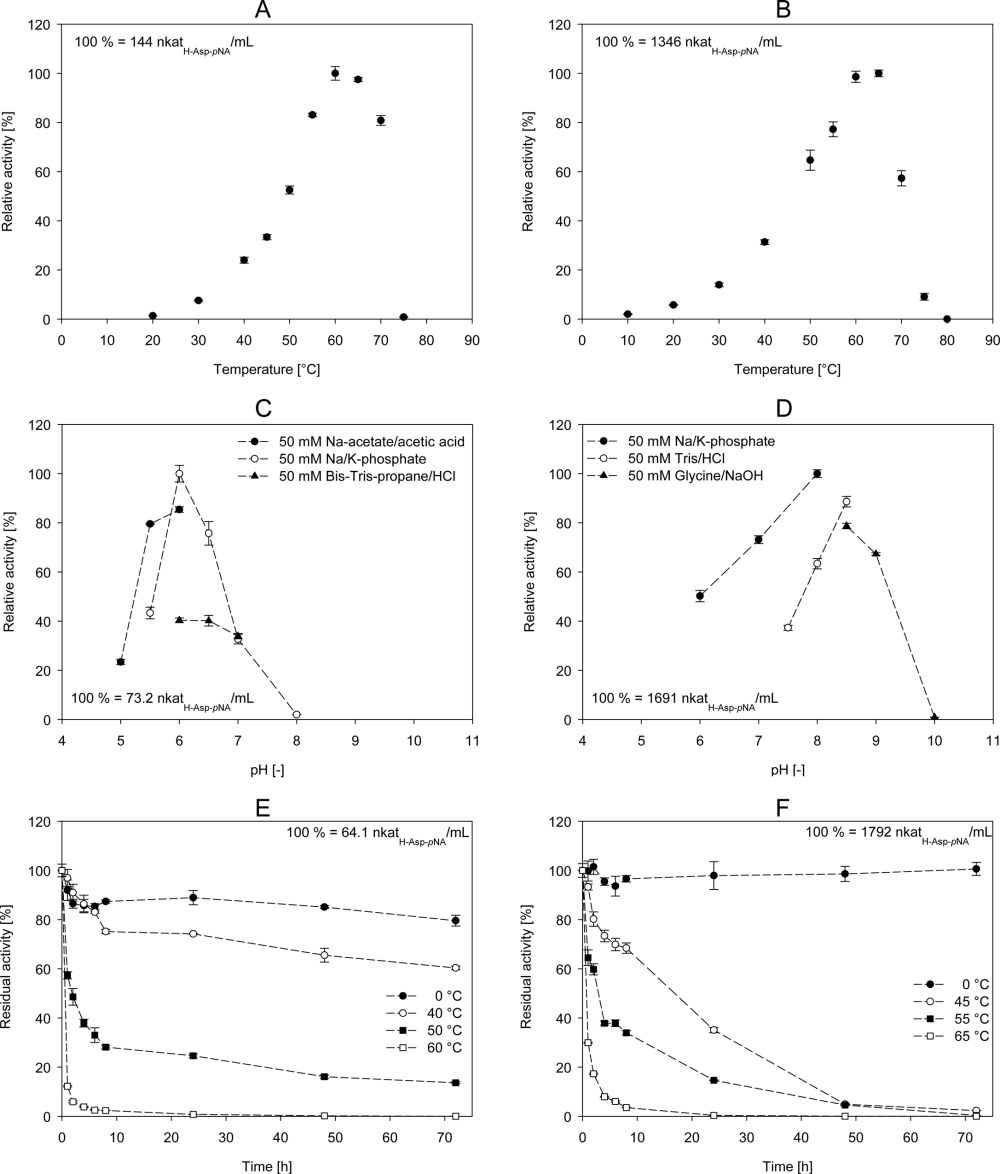
**Determination of the optimum temperature (A and B), the optimum pH (C and D) and the temperature stability (E and F) of *Lb*-PepA (A, C, D) and *Lc*-PepA (B, D, F)**. The means ± standard deviation of three independent measurements are presented.

At first, the influence of the temperature on the initial PepA activity was determined. As shown in Fig 3A, the optimum temperature for *Lb*-PepA was determined at 60°C (100% = 144 nkat_H-Asp-pNA_ mL^-1^). At a higher temperature (65°C), 97% of the maximum activity was achieved. Almost no activity (0.8%) was detected at 75°C. The highest activity for *Lc*-PepA was determined at 65°C (100% = 1345 nkat_H-Asp-pNA_ mL^-1^; Fig 3B). A minor lower activity (98%) was detected at 60°C, and a residual activity of 9% was measured at 75°C. Thus, the direct comparison of both enzymes showed a similar optimum temperature for the initial enzyme activity, whereas for *Lc*-PepA it was slightly higher.

Secondly, the optimum pH of the two different PepA enzymes was determined using different buffers (all 50 mM; Fig 3C and 3D). The highest activity for *Lb*-PepA was detected using Na/K-phosphate buffer (pH 6; 100% = 73.2 nkat_H-Asp-pNA_ mL^-1^; Fig 3C). At the same pH value, but using Na-acetate/acetic acid buffer or Bis-Tris-propane/HCl buffer, the activity was 85% and 40%, respectively. Almost no *Lb*-PepA activity (2%) was determined at a pH value of 8.0. These results differ from the pH profile of *Lc*-PepA. The highest activity for *Lc*-PepA was determined at pH 8.0 using Na/K-phosphate buffer (100% = 1691 nkat_H-Asp-pNA_ mL^-1^; Fig 3D). At a pH value of 6.0, the optimum of *Lb*-PepA, *Lc*-PepA had a residual activity of 50%. At a pH value of 10.0, using glycine/NaOH buffer, the residual activity of *Lc*-PepA was 1%.

### Temperature and storage stability of *Lb*-PepA and *Lc*-PepA

The temperature stability of *Lb*-PepA and *Lc*-PepA was determined at 0°C, as well as at the optimum temperature and 10°C and 20°C below the optimum temperature (Fig 3E and 3F). At 0°C (on ice), both enzymes were stable over the analysis time (72 h) with a residual activity of 80% and 100% for *Lb*-PepA and *Lc*-PepA, respectively. At their optimum temperatures, the stability of both enzymes was quite low and almost no activity was detectable after 24 h. In the case of *Lb*-PepA, the temperature stability at 10°C and 20°C below the optimum temperature was better compared to *Lc*-PepA. At 10°C below the optimum temperature, *Lb*-PepA had a residual activity of 14% after 72 h, whereas *Lc*-PepA showed almost no activity (0.3%). At 20°C below the optimum temperatures, the difference in stability between both enzymes was higher. After 72 h, *Lb*-PepA showed a residual activity of 60%, whereas *Lc*-PepA had only 2% of activity remaining. In addition, the temperature stability of *Lb*-PepA and *Lc*-PepA was analyzed in the presence of the optimal concentration of CoCl_2_ (see below) during the incubation at the particular temperatures. As a result, no stabilizing effect of the metal ions was determined (data not shown).

The storage stability for both PepA enzymes was tested under four different conditions ((i)–(iv), see Material and Methods for details). The storage at -80°C showed good results and a residual activity of 99% and 90% was determined for *Lb*-PepA and *Lc*-PepA, respectively, after two months. The storage stability after lyophilization and subsequent storage at 20°C in a desiccator was the lowest of all four storage conditions tested. The residual activity after two months was 62% and 20% for *Lb*-PepA and *Lc*-PepA, respectively. By contrast, the storage of the lyophilized enzyme preparations at -80°C resulted in a residual activity for *Lb*-PepA and *Lc*-PepA of 100%. Finally, the influence of freezing and thawing on the particular PepA activity was investigated. The enzyme solutions were frozen and thawed six times during the storage time of two months and showed a residual activity of 100% and 75% for *Lb*-PepA and *Lc*-PepA, respectively, at the end of the period of time.

### Influence of metal ions on the PepA activity

**Fig 4:**
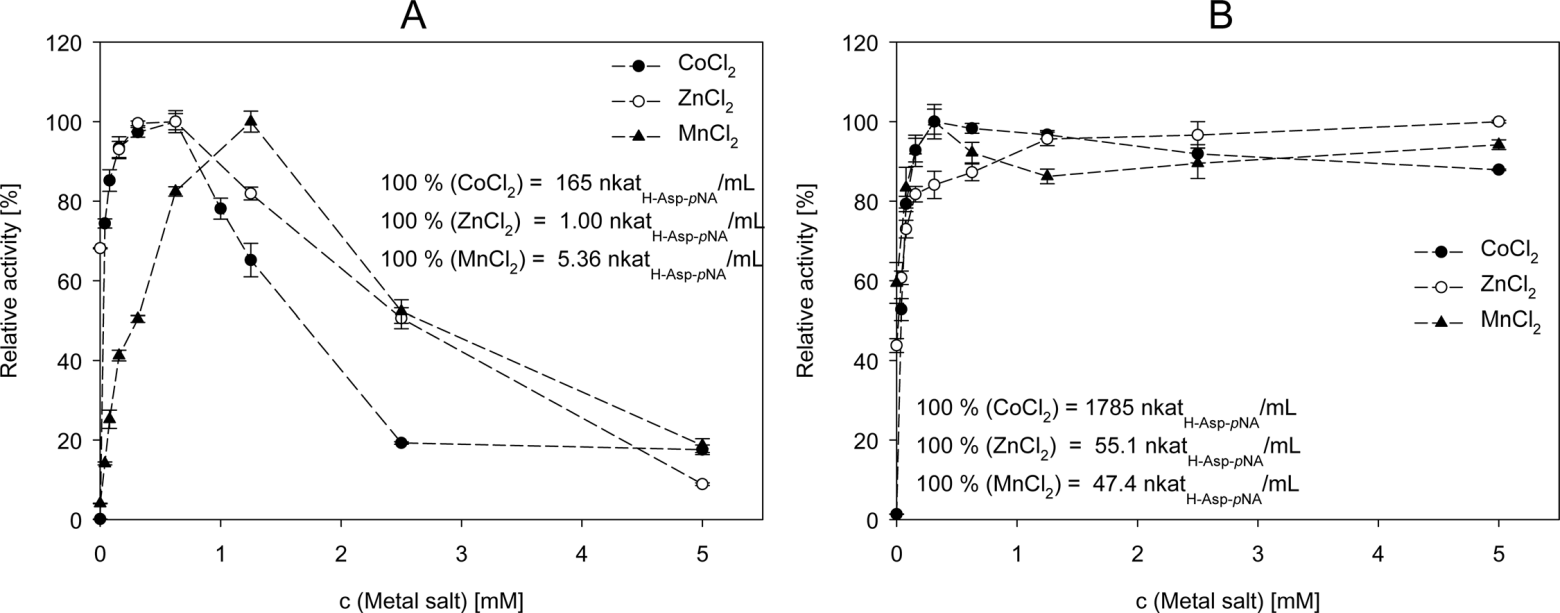
**Determination of the influence of different metal salts on the activity of *Lb*-PepA (A) and *Lc*-PepA (B).** The means ± standard **deviation of three independent measurements are presented.**

The influence of different metal ions on the particular PepA activity was investigated (Fig 4A and 4B). All metal salts were used as chlorides to prevent an influence of the anion. Without any added metal salt, the activity of *Lb*-PepA was 0.2 nkat_H-Asp-pNA_ mL^-1^ (0.12% of the overall maximum activity; Fig 4A). The highest *Lb*-PepA activity (100% = 165 nkat_H-Asp-pNA_ mL^-1^) overall was obtained with 0.625 mM CoCl_2_ added. In the case of ZnCl_2_, an addition of 0.625 mM also resulted in the highest *Lb*-PepA activity, but the activity was only 1 nkat_H-Asp-pNA_ mL^-1^. The highest activity (5.36 nkat_H-Asp-pNA_ mL^-1^) for MnCl_2_ was determined for an added concentration of 1.25 mM. The *Lb*-PepA activity decreased for all three metal salts tested with concentrations above the optimum concentration determined. The activity of *Lc*-PepA without any added metal salt was 24.1 nkat_H-Asp-pNA_ mL^-1^ (1.35% of the overall maximum activity; Fig 4B). The overall highest *Lc*-PepA activity (100% = 1785 nkat_H-Asp-pNA_ mL^-1^) was obtained with 0.3125 mM CoCl_2_ added. The highest *Lc*-PepA activity (47.4 nkat_H-Asp-pNA_ mL^-1^) for MnCl_2_ was also determined for an added concentration of 0.3125 mM. By contrast, the highest *Lc*-PepA activity (55.1 nkat_H-Asp-pNA_ mL^-1^) was obtained with an added ZnCl_2_ concentration of 5 mM. In contrast to *Lb*-PepA activity, the *Lc*-PepA activity decreased only slightly or was almost constant with metal salt concentrations above the optimum concentrations determined.

In addition, the reactivating effect of apo-PepA by five different metal salts was investigated. After treating *Lb*-PepA and *Lc*-PepA with 20 mM EDTA (ethylenediaminetetraacetic acid) and subsequent dialysis, no PepA activity was determined. CoCl_2_, ZnCl_2_, MnCl_2_, CaCl_2_ and MgCl_2_ were then added individually up to final concentrations of 5 mM each. A reactivating effect of *Lb*-PepA and *Lc*-PepA was determined for CoCl_2_, ZnCl_2_ and MnCl_2_, but none was determined for CaCl_2_ and MgCl_2_.

### Influence of organic solvents, inhibitors and other reagents on the PepA activity

The substrate and some of the reagents tested were dissolved in organic solvents prior to their addition to the assay due to their limited solubility in pure water (Table 1). The *p*NA standard assay contained 5.2% (v/v) DMSO (dimethyl sulfoxide). The activity was measured in the presence of an additional 4.2% (v/v) of the particular solvent to determine the influence of each organic solvent on the PepA activity. The activity value after the addition of 4.2% (v/v) H_2_O_dd_ was used as a reference (100%). Both the activity of *Lb*-PepA and *Lc*-PepA were reduced by additional DMSO (about 85% residual activity). However, all other solvents tested (ethanol, acetone, dimethylformamide (DMF)) reduced the particular PepA activity stronger than DMSO. Thus, DMSO is the most suitable organic solvent for substrates that are not soluble in water.

**Table 1:** Effect of various solvents, inhibitors and other reagents on the PepA activity.

	Substance	Concentration	Relative activity^1^ [%]	
			*Lb*-PepA	*Lc*-PepA
**Solvent [% (v/v)]**	Acetone	4.2	78.5	62.8
	Ethanol	4.2	83.7	74.3
	DMSO	4.2	84.9	86.1
	DMF	4.2	21.9	15.1
**Reagents [mM]**	Imidazol^2^	40	66.2	79.0
	SDS^2^	0.1	88.8	73.4
		1.0	27.1	37.1
		10	0.01	2.79
	DTT^2^	0.001	104	30.8
		0.01	94.5	13.0
		0.1	88.3	3.65
	*β*-mercaptoethanol^2^	0.01	98.7	74.4
		0.1	99.4	41.8
		1.0	96.3	3.99
	EDTA^2^	0.1	100	64.4
		1.0	0	0
	1,10-phenanthroline^3^	0.1	91.9	96.1
		1.0	78.9	0.49
		10	0	0
	PMSF^4^	0.1	90.4	91.8
		1.0	90.7	87.6
		10	65.1	68.2
	Pepstatin A^5^	0.001	99.1	93.9
		0.01	98.1	92.4
		0.1	99.1	91.7
	E64^5^	0.001	104	90.3
		0.01	104	90.0
		0.1	103	88.1

^1^The value of 100% was determined in the presence of the corresponding solvent without the additional substance.

The substances were dissolved in

^2^H_2_O_dd_

^3^Acetone

^4^Ethanol

^5^DMSO.

Presented are the means of three independent measurements and the standard deviation was < 5%.

The PepA activity values, which were measured in the presence of additional 4.2% (v/v) water, acetone, DMSO or ethanol, were considered as 100% for the inhibition studies of the different substances (Table 1). The addition of the cysteine peptidase inhibitor E64 showed no effect on *Lb*-PepA and a negligible effect on *Lc*-PepA. The same was observed for the carboxy peptidase inhibitor pepstatin A and the serine peptidase inhibitor PMSF (phenylmethylsulfonyl fluoride). The metallopeptidase inhibitor 1,10-phenantroline and the metal chelating reagent EDTA had a strong inactivating effect on both PepA enzymes. This indicates that *Lb*-PepA and *Lc*-PepA belong to the group of metallopeptidases. A difference between both enzymes was observed concerning the disulfide bond-reducing agents DTT (dithiothreitol) and *β*-mercaptoethanol. They had no effect on the *Lb*-PepA activity but inactivated *Lc*-PepA completely. This indicates that potential disulfide bonds are essential for the activity of *Lc*-PepA, but not for *Lb*-PepA.

### Determination of the substrate specificity and product inhibition of PepA

The substrate specificity of *Lb*-PepA and *Lc*-PepA was analyzed using twelve single amino acid-*p*NA substrates (see Material and Methods for details). Both PepA enzymes exhibited activity only for the substrates H-Asp-*p*NA and H-Glu-*p*NA. In the case of *Lb*-PepA, the highest activity (100% = 118 nkat mL^-1^) was determined using H-Asp-*p*NA as a substrate. The activity was 2.33% using H-Glu-*p*NA as a substrate compared to the activity determined with H-Asp-*p*NA as a substrate. The highest activity (100% = 1218 nkat mL^-1^) for *Lc*-PepA was also determined with H-Asp-*p*NA as a substrate. In contrast to *Lb*-PepA, the activity of *Lc*-PepA with H-Glu-*p*NA as a substrate was higher with 45.5%.

The product inhibition of *Lb*-PepA and *Lc*-PepA was tested for the single amino acids L-Asp and L-Glu using either H-Asp-*p*NA or H-Glu-*p*NA as a substrate (Table 2). None of the enzymes was inhibited by L-Asp up to a tested concentration of 1 mM independent of the substrate used for the PepA activity determination. Both enzymes were inhibited at an L-Asp concentration of 10 mM and showed a residual activity between 61% and 78%. There was a difference between both enzymes using the product L-Glu. The *Lb*-PepA was not inhibited in all cases, whereas *Lc*-PepA was inhibited in all combinations tested. The strongest inhibition of *Lc*-PepA (69% residual activity) was determined for a L-Glu concentration of 10 mM and H-Glu-*p*NA as a substrate.

**Table 2:** Relative PepA activity^1^^,^^2^ in the presence of potential product inhibitors.

Substrate	Product inhibitor	c(Product inhibitor) [mM]	*Lb*-PepA activity [%]	*Lc*-PepA activity [%]
**H-Asp-*p*NA**	**L-Asp**	0.1	no inhibition	no inhibition
		1.0	no inhibition	no inhibition
		10	60.5	78.2
	**L-Glu**	0.1	no inhibition	91.6
		1.0	no inhibition	89.3
		10	no inhibition	82.3
**H-Glu-*p*NA**	**L-Asp**	0.1	no inhibition	no inhibition
		1.0	no inhibition	no inhibition
		10	66.2	76.0
	**L-Glu**	0.1	no inhibition	85.3
		1.0	no inhibition	71.8
		10	no inhibition	68.6

^1^ *Lb*-PepA (100%) = 88.1 nkat_H-Asp-pNA_ mL^-1^ or 1.96 nkat_H-Glu-pNA_ mL^-1^.

^2^ *Lc*-PepA (100%) = 919 nkat_H-Asp-pNA_ mL^-1^ or 409 nkat_H-Glu-pNA_ mL^-1^.

Presented are the means of three independent measurements and the standard deviation was < 5%.

### Determination of apparent kinetic parameters of PepA

**Fig 5:**
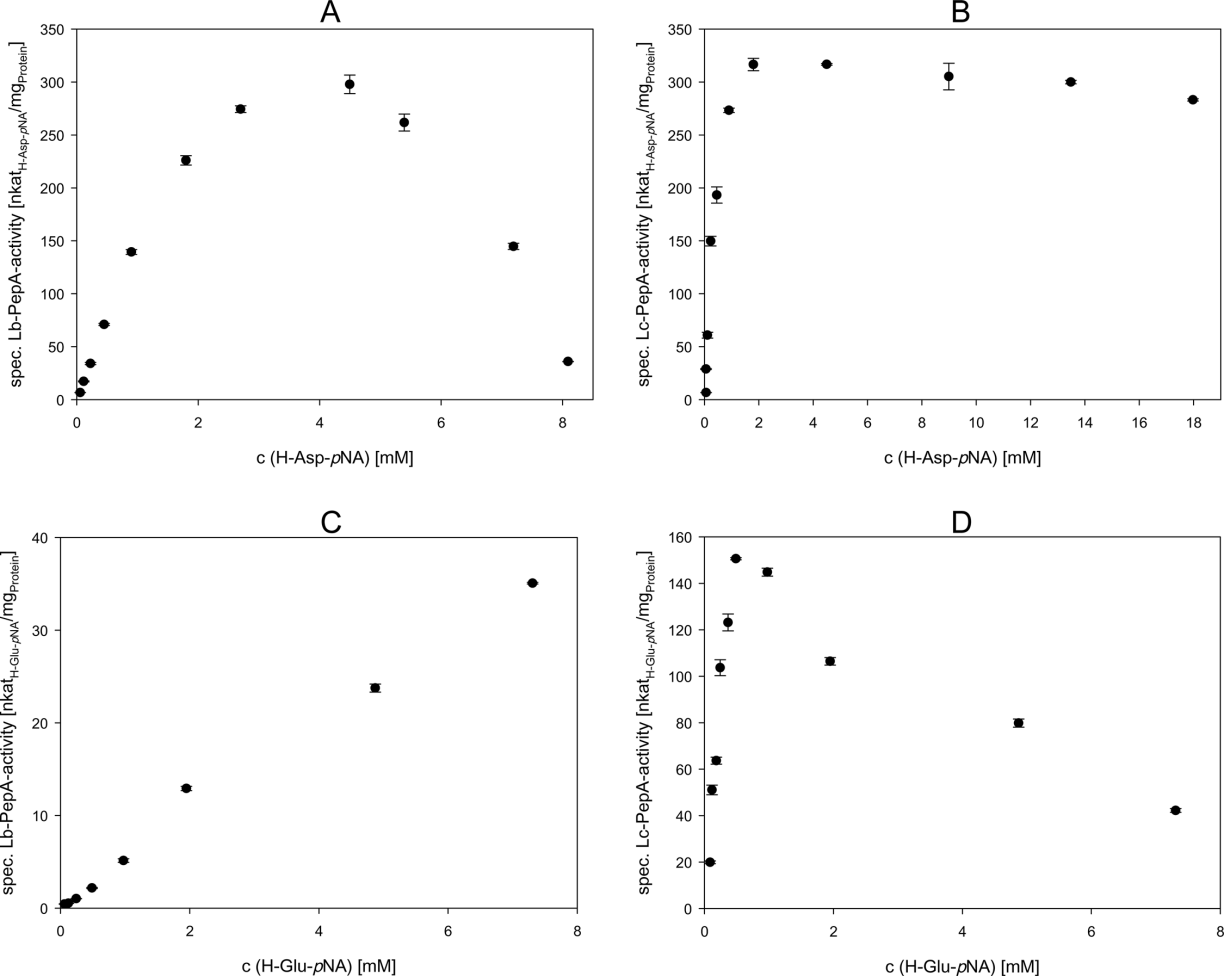
**Determination of kinetic parameters of *Lb*-PepA (A and C) and *Lc*-PepA (B and D) using H-Asp-*p*NA (A and B) and H-Glu-*p*NA (C and D) as a substrate.** The Michaelis-Menten plots are displayed and the results presented are the means ± standard deviation of three independent measurements. The calculation of the kinetic parameters were performed by nonlinear regression fitting using SigmaPlot 12.5 (Systat Software, Inc., San Jose, CA).

The apparent kinetic parameters of *Lb*-PepA and *Lc*-PepA (*V*_max_, *K*_M_ and *K*_IS_) were determined using either H-Asp-*p*NA or H-Glu-*p*NA as a substrate. The particular specific activities were plotted according to Michaelis-Menten (Fig 5) and the kinetic parameters (Table 3) were calculated by nonlinear regression fitting using SigmaPlot 12.5. A strong substrate inhibition was observed for *Lb*-PepA using the substrate H-Asp-*p*NA, as seen in Fig 5A. In the case of using H-Glu-*p*NA as a substrate (Fig 5C), no substrate inhibition was determined up to 7.3 mM. A higher concentration was not determinable due to a reduced solubility of the substrate. Based on the *K*_M_ and *V*_max_ values determined, it was shown clearly that the preferable substrate for *Lb*-PepA is H-Asp-*p*NA. A substrate inhibition was also determined for H-Asp-*p*NA as a substrate for *Lc*-PepA (Fig 5B). However, this substrate inhibition was less severe compared to the substrate inhibition of H-Asp-*p*NA for *Lb*-PepA. In contrast to *Lb*-PepA, a substrate inhibition was determined for *Lc*-PepA using H-Glu-*p*NA as a substrate (Fig 5D). This substrate inhibition was stronger for *Lc*-PepA than that using H-Asp-*p*NA as a substrate. The most preferable substrate for *Lc*-PepA was also H-Asp-*p*NA due to the comparable *K*_M_ values and the higher *V*_max_ and *K*_IS_ values.

**Table 3:** Apparent kinetic parameters of *Lb*-PepA and *Lc*-PepA using H-Asp-*p*NA or H-Glu-*p*NA as a substrate. The calculation of the kinetic parameters were performed by nonlinear regression fitting using SigmaPlot 12.5 (Systat Software, Inc., San Jose, CA).

	H-Asp-*p*NA			H-Glu-*p*NA		
	*V*_max_ [nkat mg_Protein_^-1^]	*K*_M_ [mM]	*K*_IS_ [mM]	*V*_max_ [nkat mg_Protein_^-1^]	*K*_M_ [mM]	*K*_IS_ [mM]
***Lb*-PepA**	318	1.21	6.66	154	25.2	n.a.
***Lc*-PepA**	324	0.34	41.9	154	0.19	3.61

n.a: not applicable.

Presented are the means of three independent measurements and the standard deviation was < 5%.

Finally, the apparent kinetic parameters of reactivated apo-*Lb*-PepA were determined using H-Asp-*p*NA and H-Glu-*p*NA as a substrate (Table 4). No differences were observed concerning the *K*_M_ and *K*_IS_ values between *Lb*-PepA (see above) and apo-*Lb*-PepA reactivated with CoCl_2_. The *K*_M_ and *K*_IS_ values determined for each substrate used were in the same range, independent of the metal salt used for reactivation of the apo-*Lb*-PepA. A notable difference was observed concerning the *V*_max_ values. The highest activity was always determined using CoCl_2_ for reactivation, followed by MnCl_2_ and ZnCl_2_. In the case of using ZnCl_2_ for reactivation apo-*Lb*-PepA and H-Glu-*p*NA as a substrate, the *Lb*-PepA activity determined was too low to confidently evaluate the kinetic parameters.

**Table 4:** Apparent kinetic parameters of reactivated apo-*Lb*-PepA using H-Asp-*p*NA or H-Glu-*p*NA as a substrate. The calculation of the kinetic parameters were performed by nonlinear regression fitting using SigmaPlot 12.5 (Systat Software, Inc., San Jose, CA).

	H-Asp-*p*NA			H-Glu-*p*NA		
Metal ion	*V*_max_ [nkat mg_Protein_^-1^]	*K*_M_ [mM]	*K*_IS_ [mM]	*V*_max_ [nkat mg_Protein_^-1^]	*K*_M_ [mM]	*K*_IS_ [mM]
**Co^2+^**	198	1.14	5.69	79.8	23.1	n.a.
**Zn^2+^**	0.89	0.52	4.93	not evaluable due to low activity		
**Mn^2+^**	1.98	0.62	5.17	2.96	19.5	n.a.

n.a: not applicable.

Presented are the means of three independent measurements and the standard deviation was < 5%.

## Discussion

In this work, the putative *pepA* gene from *Lb*. *delbrueckii* ssp. *lactis* DSM 20072 was cloned into the vector pET20b(+) and expressed using the expression host *E*. *coli* BL21(DE3). For direct comparison of the biochemical characteristics of *Lb*-PepA to the lactococcal PepA (*Lc*-PepA) from *Lc*. *lactis* ssp. *lactis* DSM 20481 was also cloned and biochemically characterized.

### Structural comparison of PepA

The metal binding site of PepA from *Streptococcus pneumonia* R6 (*Sc*-PepA) was described by Kim et al. [22]. The authors stated in this article that *Sc*-PepA belongs to the M42 family of peptidases and exhibits a dodecameric structure. Although the presence of the zinc ions in the crystal was not confirmed, they modeled two zinc ions in the active site based on the electron densities of its crystal diffraction. His66 and Asp236 coordinated one of the zinc ions, whereas Glu214 and His318 coordinated the second zinc ion. Asp181 coordinated both zinc ions. The substrate binding pocket itself was constructed by Asp236, Ser238, Leu255, Arg257, Thr309, and Gly311. The authors stated that Arg257 is notable because the position of the Arg257 side chain creates a positive patch in the S1 pocket and, therefore, the positive patch of *Sc*-PepA appears to be responsible for its specificity towards acidic amino acids in the S1 position.

Due to the well conserved metal coordinating residues in the active site [22], novel PepA enzymes can be found, and a comparison to the known PepA enzymes from *Lc*. *lactis* sp. and the novel PepA from *Lb*. *delbrueckii* is shown in Table 5. Although the gene and protein homology is quite low compared to other PepA, the position of the catalytically important residues appears similar. This indicates that the different enzymes show the same catalytic activity, but, due to the low homology, other enzyme characteristics, such as optimum conditions and stability, could differ among the PepA enzymes.

**Table 5:** Comparision of the *pepA* gene and PepA protein from different microorganisms.

	*Lc*. *lactis* ssp. *cremoris* MG1363	*Lc*. *lactis* ssp. *lactis* CV56^2^	*Lb*. *delbrueckii* ssp. *lactis* DSM 20072	*St*. *pneumoniae* R6
**UniProt ID**	Q48677	F2HIS5	F0HXE4	Q8DNJ7
***pepA* gene [bp]**	1068	1068	1086	1065
**PepA protein [aa/kDa]**	355/38.32	355/38.34	361/40.03	354/38.02
**Gene homology^1^ [%]**	100	85	26	38
**Protein homology^1^ [%]**	100	94	30	60
**Active residue (Glu; proton acceptor)**	213^5^	213^3^	215^3^	213^3^
**Residue to create a positive patch (Arg)**	258^5^	258^5^	258^5^	257^4^
**Metal binding site:**				
His	65^5^	65^5^	67^5^	66^4^
Asp	181^5^	181^5^	181^5^	181^4^
Glu	214^5^	214^5^	216^5^	214^4^
Asp	236^5^	236^5^	232^5^	236^4^
His	319^5^	319^5^	324^5^	318^4^

^1^ The pepA gene/PepA protein from *Lc*. *lactis* ssp. *cremoris* MG1363 was used as a reference.

^2^ The *pepA* gene is identical to the *pepA* gene from *Lc*. *lactis* ssp. *lactis* DSM 20481 used in this study.

^3^ Automated UniRule annotation.

^4^ Experimental evidence [22].

^5^ By similarity.

### Classification of aminopeptidase A

The name aminopeptidase A (PepA) is confusing. The aspartyl aminopeptidase (EC 3.4.11.21) and the glutamyl aminopeptidase (EC 3.4.11.7) are both called aminopeptidase A (http://www.brenda-enzymes.org). Both enzyme classes catalyze the release of N-terminal Asp or Glu from a substrate. However, aspartyl aminopeptidase was described from eukaryotic sources and belongs to the M18 family of peptidases, whereas glutamyl aminopeptidase was especially described for milk-associated microorganisms and belongs mainly to the M42 family of peptidases (http://www.brenda-enzymes.org). Unfortunately, there is a third enzyme class (leucyl aminopeptidase; EC 3.4.11.10) which is also called aminopeptidase A. This enzyme class releases N-terminal amino acids, preferentially Leu, but not Asp or Glu (http://www.brenda-enzymes.org). Thus, although *Lb*-PepA and *Lc*-PepA have a higher activity against the substrate H-Asp-*p*NA they have to be classified as glutamyl aminopeptidases (EC 3.4.11.7).

### Comparison of biochemical characteristics of different PepA

In 1987, Exterkate and De Veer [11] purified and characterized the first PepA of a LAB, called *Streptococcus cremoris* HP, which is now called *Lc*. *lactis* ssp. *cremoris* HP. Four years later, Niven [19] described the characteristics of PepA from *Lc*. *lactis* ssp. *lactis* NCDO 712, followed by Bacon et al. [20], who examined the PepA from *Lc*. *lactis* ssp. *cremoris* AM2. All these PepA enzymes belonged to microorganisms of the genus *Lactococcus*. In the current study, the first PepA from a microorganism of the genus *Lactobacillus* is described. Some selected characteristics of PepA from different microorganisms are shown in Table 6. The optimum temperature of all PepA described varied between 50 and 65°C. The optimum pH for all lactococcal PepA was between 8.0 and 8.3, and only the novel PepA from *Lb*. *delbrueckii* had an acidic pH optimum (pH 6.0). In agreement with our results, the lactococcal PepA was inhibited by DTT, but not the PepA from *Lb*. *delbrueckii*. To the best of our knowledge, no crystal structures are available yet, neither for *Lb-PepA* nor *Lc*-PepA. Thus, further research is needed to explain the difference concerning the inhibition by disulfide bond-reducing agents.

**Table 6:** Summary of selected characteristics of PepA from different microorganisms.

PepA source	Optimum Temp. [°C]	Optimum pH [–]	Inhibitors	Product inhibition	Reference
*Lb*. *delbrueckii* ssp. *lactis* DSM 20072	60	6.0	1,10-phenantroline, EDTA	L-Asp (10 mM)	Current study
*Lc*. *lactis* ssp. *lactis* DSM 20481	65	8.0	1,10-phenantroline, EDTA; DTT, *β*-mercaptoethanol	L-Asp (10 mM), L-Glu (0.1 mM)	Current study
*Lc*. *lactis* ssp. *cremoris* HP	50–55	n.d.	1,10-phenantroline, EDTA; DTT	n.d.	[11]
*Lc*. *lactis* ssp. *cremoris* AM2	n.d.	8.3	1,10-phenantroline, EDTA	n.d.	[20]
*Lc*. *lactis* ssp. *lactis* NCDO 712	65	8.0	EDTA	n.d.	[19]

n.d.: not determined.

### Potential application of PepA for food protein hydrolysis using an enzyme membrane reactor

As mentioned in the introduction, PepA can probably be used for the production of flavoring hydrolysates out of glutamyl/aspartyl-rich food proteins. Nowadays, the industrial biotransformation of proteins is mainly performed in discontinuous batch processes [30,31]. A promising alternative to the batch processes are continuous biotransformations using an enzyme membrane reactor [30,32]. Using this process approach, the enzymes are located in a reaction space, entrapped by a membrane and, thus, can be reused, which is a remarkable economic benefit [30,33]. Due to the high molecular mass of the PepA enzymes (about 480 kDa), they will not penetrate the membrane used (normally 1–10 kDa). The sufficient temperature stability, especially of *Lb*-PepA, also makes this enzyme interesting for application in an EMR.

In conclusion, the gene sequence for the PepA from *Lb*. *delbrueckii* ssp. *lactis* DSM 20072, heretofore described as putative, was heterologously expressed in *E*. *coli* and the recombinant protein showed the enzyme activity desired. Thus, the gene/function relationship was proven and the *Lb*-PepA produced was characterized biochemically for the first time. Most of the characteristics determined were different to the PepA enzymes from *Lactococcus* sp. described heretofore. The more acid optimum pH value of *Lb*-PepA (pH 6.0) compared to *Lc*-PepA (pH 8.0) makes the *Lb*-PepA especially interesting for food protein hydrolysis, because food generally has an acid or neutral pH value. In addition, *Lb*-PepA showed higher temperature stability and was not product-inhibited by L-Glu. Due to the fact that the *Lb*-PepA was characterized using synthetic *p*NA-substrates, as is common for this enzyme class in the literature, a closing statement about its activity against original peptide substrates cannot be made at this point and was not in the focus of this fundamental study. The activity against original peptide substrates and the application of *Lb*-PepA in food protein hydrolysis will be presented in a further study.
